# NIFTP-adjusted risk estimation of Bethesda thyroid cytology categories should consider the indication for FNA according to TIRADS

**DOI:** 10.1007/s12020-024-03800-9

**Published:** 2024-04-03

**Authors:** Andrea Leoncini, Chiara Camponovo, Elena Gamarra, Tommaso Piticchio, Lorenzo Ruinelli, Mario Rotondi, Vito Cantisani, Giorgio Treglia, Pierpaolo Trimboli

**Affiliations:** 1 Clinic for Radiology and Interventional Radiology, Imaging Institute of Southern Switzerland, Ente Ospedaliero Cantonale (EOC), Lugano, Switzerland; 2 Clinic for Endocrinology and Diabetology, Ente Ospedaliero Cantonale (EOC), Lugano, Switzerland; 3 Endocrinology Section, Department of Clinical and Experimental Medicine, Garibaldi Nesima Hospital, University of Catania, Catania, Italy; 4 Team Data Science & Research, Area ICT, Ente Ospedaliero Cantonale (EOC), Bellinzona, Switzerland; 5 Clinical Trial Unit, Ente Ospedaliero Cantonale (EOC), Bellinzona, Switzerland; 6 Department of Internal Medicine and Therapeutics, University of Pavia, Pavia, Italy; 7 Unit of Endocrinology and Metabolism, Laboratory for Endocrine Disruptors, Istituti Clinici Scientifici Maugeri IRCCS, Pavia, Italy; 8 Department of Radiological and Oncological Sciences and Pathological Anatomy, “Sapienza” University of Rome, Rome, Italy; 9 Clinic of Nuclear Medicine, Imaging Institute of Southern Switzerland, Ente Ospedaliero Cantonale (EOC), Bellinzona, Switzerland; 10 Faculty of Biology and Medicine, University of Lausanne, Lausanne, Switzerland; 11 Faculty of Biomedical Sciences, Università Della Svizzera Italiana, Lugano, Switzerland

**Keywords:** Thyroid nodule, NIFTP, TIRADS, Ultrasound, Bethesda

## Abstract

**Background:**

Non-invasive follicular thyroid neoplasm with papillary-like nuclear features (NIFTP) was firstly described in 2016. Since NIFTP is thought a non-malignant tumor, the Bethesda system for thyroid cytology proposes two estimations of risk of malignancy of the diagnostic categories, one considering NIFTP as cancer and another one considering it as a benign neoplasm. The present study aimed to review NIFTPs in a single center, re-assess them across categories of three Thyroid Imaging Reporting and Data Systems (TIRADSs), and define the indication for biopsy according to the category-specific size cut-offs.

**Methods:**

The study period was from 2017 to 2023. The institutional database was searched for histologically proven NIFTPs with preoperative ultrasound images. NIFTPs were re-assessed according to the American College of Radiology (ACR), European (EU), and Korean (K) TIRADSs. The indication for biopsy was defined according to TIRADS category-specific size threshold.

**Results:**

Twenty NIFTPs from 19 patients were included. The median size of the NIFTPs was 23 mm. According to ultrasound, 80–85% of NIFTPs were at low-intermediate risk and 5–15% at high risk without significant difference among the tree TIRADSs (*p* = 0.91). The indication for FNA, according to three TIRADSs, was found in 52–58% of cases with no significant difference among systems (*p* = 0.96).

**Conclusion:**

NIFTPs have heterogeneous presentation according to TIRADSs with very low indication rate for FNA.

## Introduction

Non-invasive follicular thyroid neoplasm with papillary-like nuclear features (NIFTP) is a histological entity first described in 2016 [1, 2]. Since its initial description, many papers have investigated its prevalence among all thyroid tumors, and among all thyroid nodules (TNs) undergoing fine-needle aspiration (FNA). The evidences show that NIFTPs can be found in all cytological categories with the highest prevalence among cytological indeterminate ones, such as category III and IV of the Bethesda System for Reporting Thyroid Cytopathology (TBSRTC) [3]. Given that NIFTP is though having indolent non-malignant/non-invasive behavior, the second edition of TBSRTC [4] included two different estimations of risk of malignancy (RoM) associated with the diagnostic categories: one considering NIFTP as cancer and another considering NIFTP as benign neoplasm. Accordingly, the recent third edition of TBSRTC [5] reported an updated NIFTP-adjusted RoM of the cytological categories from I to VI, as illustrated in Table 1.

**Table 1:** Risk of malignancy (RoM) associated with the Bethesda System for Reporting Thyroid Cytopathology (TBSRTC) categories with and without NIFTP

TBSRTC category	Original RoM, mean % (range)	RoM considering NIFTP as benign	
		RoM decrease, mean % (range)	NIFTP-adjusted RoM, %
I Nondiagnostic	14 (0–33)	1.3 (0–2)	12
II Benign	6 (0–27)	2.4 (0–4)	2
III Atypia of undetermined significance	28 (11–54)	6.4 (6–20)	16
IV Follicular neoplasm	50 (28–100)	7.1 (0.2–30)	23
V Suspicious for malignancy	81 (40–100)	9.1 (0–40)	65
VI Malignant	98 (86–100)	2.6 (0–13)	94

The table reports the estimations reported in the third edition of TBSRTC [5]

The honorable aim of presenting different RoM according to NIFTP was to reduce the resection rate of TNs patients, especially those with indeterminate FNA reports, and avoid as much as possible the postoperative radioiodine treatment. However, NIFTP can only be diagnosed on surgical specimens, and its identification by FNA not feasible in a reliable way [6]. Thus, whether NIFTP truly affects the clinical management of TNs has still to be proven.

Besides the above issues, the true impact of NIFTP in clinical practice has been investigated by authors who focused their studies on its presentation at ultrasound (US). Several papers have been published and the majority of them reported that NIFTP presents at US as low- to intermediate-risk TNs, with only a minority assessed at high risk/suspicion. These studies included series of histologically proven NIFTPs that were retrospectively re-assessed according to US-based risk stratification systems often reported with the acronym TIRADS (Thyroid Imaging Reporting and Data System). Table 2 summarizes the characteristics and results of studies evaluating the NIFTP assessment across TIRADS classes [7–17].

**Table 2:** Characteristics and results of studies evaluating the NIFTP presentation according to TIRADSs

First author (year)	NIFTPs (*n*)	Size	US-based risk category
Matrone [7]	116	Median 21 (IQR 13–37) mm	ACR-TIRADS 1, 1.7% ACR-TIRADS 2, 2.6% ACR-TIRADS 3, 55.2% ACR-TIRADS 4, 40.5%
			EU-TIRADS 2, 5.2% EU-TIRADS 3, 57.8% EU-TIRADS 4, 28.4% EU-TIRADS 5, 8.6%
			K-TIRADS 2, 5.2% K-TIRADS 3, 57.8% K-TIRADS 4, 15.3% K-TIRADS 5, 1.7%
			ATA very low suspicion, 4.3% ATA low suspicion, 58.6% ATA intermediate suspicion, 29.3% ATA high suspicion, 7.8%
			AACE/ACE/AME low risk, 6% AACE/ACE/AME intermediate risk, 86.2% AACE/ACE/AME high risk, 7.8%
Alzumaili [8]	183	Median 23 (min-max 3–75) mm	ACR-TIRADS 1, 1% ACR-TIRADS 2, 3% ACR-TIRADS 3, 26% ACR-TIRADS 4, 50% ACR-TIRADS 5, 20%
Ni [9]	29	Mean 29.9 ± 14.7 mm	ACR-TIRADS 3, 10% ACR-TIRADS 4, 16% ACR-TIRADS 5, 3%
			ATA low suspicion, 11% ATA intermediate suspicion, 14% ATA high suspicion, 4%
Taneja [10]	79	Mean 24 (min-max 1.5–80) mm	ACR-TIRADS 1, 0% ACR-TIRADS 2, 2.6% ACR-TIRADS 3, 55.3% ACR-TIRADS 4, 36.8% ACR-TIRADS 5, 5.3%
Vignali [11]	451	Median 17 (IQR 9–30) mm	EU-TIRADS 2, 3.5% EU-TIRADS 3, 96.5%
Liu [12]	30	Median 19 (min-max 3–61) mm	ACR-TIRADS 2, 20% ACR-TIRADS 3, 26.7% ACR-TIRADS 4, 40% ACR-TIRADS 5, 13.3%
			ATA very low suspicion, 3.3% ATA low suspicion, 43.3% ATA intermediate suspicion, 12.4% ATA high suspicion, 13.3%
Boursier [13]	14	Mean 30 ± 15.8 mm	TIRADS 2, 15.4% TIRADS 3, 46.2% TIRADS 4a, 15.4% TIRADS 4b, 23.1%
Larouche [14]	44	Mean 22.9 ± 12.3 mm	ACR-TIRADS 1–3, 45.4% ACR-TIRADS 4, 54.6%
Yang [15]	76	Mean 22 (min-max 10–56) mm	ACR-TIRADS 3, 26.3% ACR-TIRADS 4, 73.7%
Lee [16]	20	Mean 25 (min-max 5–60) mm	K-TIRADS 2, 1.6% K-TIRADS 3, 47.5% K-TIRADS 4, 41% K-TIRADS 5, 9.8%
Rosario [17]	28	NA	ACR-TIRADS 3, 28.5% ACR-TIRADS 4, 67.8% ACR-TIRADS 5, 3.5%

Here are reported data of NIFTP distribution according to American College of Radiology (ACR)-TIRADS [7, 8, 12, 14, 15, 17], European (EU)-TIRADS [7, 11], Korean (K)-TIRADS [7, 16, 17], American Thyroid Association (ATA) risk stratification system [7, 9, 12], American Association of Clinical Endocrinology (AACE)/American College of Endocrinology (ACE)/Associazione Medici Endocrinologi (AME) risk stratification system [7], and Horvath TIRADS [13]

Based on the figures recorded by these TIRADS studies, one would ask whether the double estimation of RoM proposed by TBSRTC according to NIFTP is correct or not. In fact, this RoM estimation derives from data collected in studies searching for the prevalence of NIFTP in retrospective histological series. However, these findings do not consider that TNs are managed in clinical practice according to several features, first of all the US presentation and TIRADS risk assessment. In addition, remarkably, the indication or not for FNA is recommended/suggested according to the TIRADS category-specific size threshold. Thus, at least theoretically, we should confute the TBSRTC’s estimate because we cannot affirm that all NIFTPs would undergo FNA.

According to the above relevant clinical issues, the present study was undertaken to retrospectively review NIFTPs recorded in one single institution, re-assess them across TIRADS categories, and define the indication for FNA according to the category-specific size.

## Material and methods

### Setting

Our institution is the public health institution of our region and performs the highest number of thyroid surgeries in that region. The institutional database includes records adequate to respond to the study’s aim.

### Case selection

The study period was from January 2017 to December 2023. According to the study aim, the institutional database was searched for patients with histologically proven NIFTP. As an inclusion criterion, preoperative US images were available in RIS-PACS. Histological and ultrasonographic data of included patients were matched. All NIFTPs were re-assessed according to American College of Radiology (ACR)-TIRADS [18], European (EU)-TIRADS [19], and Korean (K)-TIRADS [20] by expert US operators. NIFTP size was used to establish the indication for FNA.

### Measures and reference standard

The ultrasonographic RoM of NIFTPs was defined according to three TIRADSs [18–20]. The indication for FNA was assessed according to the size threshold associated with any TIRADS category [18–20]. Histology was adopted as the gold standard of the study.

### Statistical analysis

Continuous parameters were reported in the manuscript as median and interquartile range (IQR). Frequencies between subgroups were compared using chi-square test. The statistical significance level was set at *p* < 0.05. Statistical analyses were conducted with the software GraphPad Prims version 7 (GraphPad software, CA, USA).

### Ethics

This study was approved by the local Ethics Committee, and patients gave informed consent for the study.

## Results

### Demographic and histological data

According to the selection criteria, 23 NIFTPs from 21 patients were initially found in the institutional database. One patient refused to be enrolled. After removing three cases in which it was not possible to match the US and histological data, the study series included 19 NIFTPs from 19 patients. There were 15 females and four males. The median age was 60 (53–67) years. The median size of the NIFTPs was 23 (10–46) mm. Among the 19 included NIFTPs, nine (47.3%) underwent FNA before surgery: five had cytological benign report, two were indeterminate, one was suspicious for malignancy, and the remaining one was read as malignant.

### NIFTP assessment across TIRADS categories

When NIFTPs were re-assessed at US, we observed that: 1) the nodules were classified as intermediate category in 63.2% of cases according to ACR-TIRADS, 47.4% according to EU-TIRADS, and 47.4% according to K-TIRADS; 2) high-risk category was assigned in 5.3%, 15.8%, and 10.5% of NIFTPs according to ACR-, EU-, and K-TIRADS, respectively; 3) the remaining cases were classified at low risk. No significant difference was found when comparing the three TIRADSs (*p* = 0.91). Table 3 details the findings of the distribution of NIFTPs across the TIRADSs categories.

**Table 3:** NIFTP assessment across the ACR-, EU, and K-TIRADS categories, and cases with indication for FNA

TIRADS	Category	Cases, *n* (%)	Indication for FNA, *n* (%)
ACR	2	2 (10.53)	0 (0)
	3	4 (21.05)	2 (10.53)
	4	12 (63.16)	8 (42.11)
	5	1 (5.26)	1 (5.26)
EU	2	2 (10.53)	0 (0)
	3	5 (26.32)	2 (10.53)
	4	9 (47.37)	6 (31.58)
	5	3 (15.79)	2 (10.53)
K	2	2 (10.53)	0 (0)
	3	6 (31.58)	2 (10.53)
	4	9 (47.37)	7 (36.84)
	5	2 (10.53)	2 (10.53)

All percentages are calculated according to the total number of 20 NIFTPs. The indication for FNA was assessed according to NIFTP’s size as recommended by American College of Radiology (ACR)-, European (EU)-, and Korean (K)-TIRADS

### Indication for FNA

**Fig. 1:**
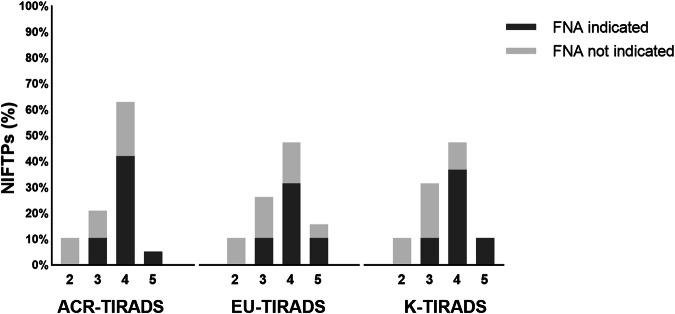
Distribution of NIFTPs across the American College of Radiology (ACR)-TIRADS, European (EU)-TIRADS, and Korean (K)-TIRADS categories and their indication or not for FNA. The values are expressed as percentage of cases among all NIFTPs

The indication for FNA was found in 57.9%, 52.6%, and 57.9% of cases according to ACR-, EU-, and K-TIRADS, respectively. The category with a higher FNA indication rate was the intermediate one with 42.1%, 31.6%, and 36.8%, respectively. No significant difference was found when comparing the indication for FNA according to the three TIRADSs (*p* = 0.96). Figure 1 summarizes the percentage distribution of NIFTPs across the TIRADSs categories and their indication or not for FNA.

## Discussion

Since TNs are a common entity with an expected low frequency of cancer, careful management of these patients is needed. In this regard, US represents the first-line diagnostic procedure due to its high reliability to stratify the risk of malignancy of TNs we face during clinical practice. More recently, the use of TIRADS has been largely and rapidly diffused with excellent results [21, 22]. The TIRADSs were undertaken with several objectives: 1) to establish a US standard lexicon, 2) to define US risk features, 3) to assess TNs across risk categories, 4) to carefully select TNs for FNA, avoiding as much as possible unnecessary biopsies. Considering that TNs can be observed in up to 70% of adults, and taking into account that less than 3–5% of them are a cancer [23], the latter represents a pivotal point. The literature showed that TIRADSs allow to reduce unnecessary FNA, even if with different performances when compared to each other [24]. With these premises, the double risk estimation proposed by TBSRTC, seeing or not NIFTP as a malignant entity should be challenged also considering the impact of TIRADS-guided management of patients. Here, we revised our institutional database to assess NIFTPs across the ACR-, EU-, and K-TIRADS categories, and analyze the rate of FNA indicated according to the TIRADSs. These results merit full discussion.

First, only a minority of NIFTPs were ultrasonographically assessed as high-risk. On the contrary, the most part of them was classified at low-to-intermediate risk of malignancy (i.e., TIRADS category from 2 to 4). This is perfectly in line with the data recorded in other studies (see Table 2). This means that NIFTP has a heterogeneous US presentation but is usually ultrasonographically unsuspicious. These findings corroborate that US features, and TIRADSs of course, are reliable to detect papillary thyroid carcinoma while their accuracy is lower for other histological types [25, 26]. The present data can contribute to create the international TIRADS endorsed by major societies [27].

Second, FNA was indicated only in just above a half of NIFTPs. This is a piece of novel information in the literature that can modify our view on the matter of NIFTP; a critical discussion is then needed. In fact, based on this data, the NIFTP-adjusted RoM estimation of FNA categories of TBSRTC should not be reliable. Since the TNs dimensional threshold indicates FNA and varies according to the TIRADS’s category (i.e., the higher the US-based risk, the lower the size to recommend FNA), the low rate of FNA indication among NIFTPs was certainly influenced by the high call rate of low-to-intermediate risk TIRADS classes. In addition, the median size of NIFTPs of the present series was just above 2 cm, again in line with the literature (see Table 2). Taking into account that about 80–85% of NIFTPs were not at high risk according to TIRADS, and considering that FNA is indicated in TNs assessed as low-risk categories of TIRADSs only when they are sized above 2–2.5 cm, at least a half of NIFTP should not receive FNA in clinical practice.

Third, according to present data and previous reports [7–17], NIFTPs have no typical US presentation. This means that we cannot identify NIFTP at US, as well as on FNA specimens indeed [6]. Thus, the present findings, even if collected retrospectively, achieve high interest for clinical practice.

Fourth, TNs patients are usually managed according to their clinical features (e.g., gender, age, comorbidities, familiarity, medications, compressive/cosmetic complaints, anxiety, and other individual characteristics). Even if we did not collect full data about our patient’s clinical characteristics, a clinically oriented TNs management should be considered also when speaking about NIFTP. Then, discovering NIFTP in histopathology, especially when it is incidentally found, should not modify our clinical practice for indicating or not for FNA.

Some limitations of the present study should be addressed. 1) This is a retrospective study, and the patients included were managed during clinical practice according to several aspects (i.e., goiter-related symptoms, TIRADS assessment, FNA indication, age, comorbidities, and others). Then, a possible selection bias is present. 2) The sample size is not large and some NIFTPs were excluded because it was not possible to match US and histological data. However, the results observed were perfectly in line with those found in other studies with larger sample size. Then, the series is reliable. On the other hand, this study presents at least two important strengths: 1) The present study aimed to analyze the indication for FNA in the attempt to confute the double RoM estimation of cytological categories proposed by TBSRTC [4, 5]. This allowed to achieve a novel information in the literature; 2) It is worth to be emphasized that all NIFTPs included were diagnosed during clinical practice. This is a crucial point because the diagnosis of NIFTP is quite difficult in histological samples prepared before the advent of this pathological entity [3].

In conclusion, the present study shows that NIFTPs have heterogeneous US presentation according to the TIRADSs with the highest prevalence of low-to-intermediate risk categories. In addition, as a novelty in the literature, the indication rate for FNA of NIFTP is very low. This means that the estimation of TBSRTC should need to be revised.
